# The Efficacy of Tetracyclines in Peripheral and Intracerebral Prion Infection

**DOI:** 10.1371/journal.pone.0001888

**Published:** 2008-03-26

**Authors:** Ada De Luigi, Laura Colombo, Luisa Diomede, Raffaella Capobianco, Michela Mangieri, Claudia Miccolo, Lucia Limido, Gianluigi Forloni, Fabrizio Tagliavini, Mario Salmona

**Affiliations:** 1 Department of Molecular Biochemistry and Pharmacology, Istituto di Ricerche Farmacologiche “Mario Negri”, Milano, Italy; 2 Department of Neuroscience, Istituto di Ricerche Farmacologiche “Mario Negri”, Milano, Italy; 3 Fondazione I.R.C.C.S. Istituto Neurologico “Carlo Besta”, Milano, Italy; National Institutes of Health, United States of America

## Abstract

We have previously shown that tetracyclines interact with and reverse the protease resistance of pathological prion protein extracted from scrapie-infected animals and patients with all forms of Creutzfeldt-Jakob disease, lowering the prion titre and prolonging survival of cerebrally infected animals. To investigate the effectiveness of these drugs as anti-prion agents Syrian hamsters were inoculated intramuscularly or subcutaneously with 263K scrapie strain at a 10^−4^ dilution. Tetracyclines were injected intramuscularly or intraperitoneally at the dose of 10 mg/kg. A single intramuscular dose of doxycycline one hour after infection in the same site of inoculation prolonged median survival by 64%. Intraperitoneal doses of tetracyclines every two days for 40 or 44 days increased survival time by 25% (doxycycline), 32% (tetracycline); and 81% (minocycline) after intramuscular infection, and 35% (doxycycline) after subcutaneous infection. To extend the therapeutic potential of tetracyclines, we investigated the efficacy of direct infusion of tetracyclines in advanced infection. Since intracerebroventricular infusion of tetracycline solutions can cause overt acute toxicity in animals, we entrapped the drugs in liposomes. Animals were inoculated intracerebrally with a 10^−4^ dilution of the 263K scrapie strain. A single intracerebroventricular infusion of 25 µg/ 20 µl of doxycycline or minocycline entrapped in liposomes was administered 60 days after inoculation, when 50% of animals showed initial symptoms of the disease. Median survival increased of 8.1% with doxycycline and 10% with minocycline. These data suggest that tetracyclines might have therapeutic potential for humans.

## Introduction

Transmissible spongiform encephalopathies are fatal neurodegenerative diseases that cause extensive loss of cerebral neurons with formation of vacuoles, giving a “sponge-like” appearance to the tissue. They include several diseases with different causes (infectious/iatrogenic, sporadic or genetic origin), recognized both in humans and animals as variations of the same disorder [1], [2].

Although the precise pathogenic mechanism is still not understood, prion diseases involve the deposition of aggregates of disease-related isoforms (PrP^Sc^) of a host-encoded protein (PrP^C^) in the central nervous system. During the progression of the disease, a portion of the α-helix and random coil structure in PrP^C^ is refolded into a β-pleated sheet in PrP^Sc^, a conformational change that renders PrP^Sc^ poorly soluble and resistant to protease digestion [3]. Consequently, PrP^Sc^ aggregates accumulate around neurons in affected brain areas, a process thought to lead to neuronal dysfunction and death, and subsequently the clinical symptoms of infection.

Many classes of molecules have been screened in experimental therapy [4]–[7], some showing promising anti-prion activity, but most of them suffer the limitation of poor passage through the blood-brain barrier (BBB) and severe toxicity. Currently, there is no therapy for prion diseases in humans. Three drugs, pentosan polysulfate [8], [9], quinacrine [10], [11] and flupirtrine [12], are currently used in the clinical management of human prion diseases, although none of them have yet been proved to be effective, reinforcing the importance of identifying new treatment strategies.

Based on structural analogies with Congo red, tetrapyrroles and acridine derivatives we hypothesized that tetracyclines might interact with PrP^Sc^ and interfere with PrP amyloid formation [13]. These old drugs have well-characterized pharmacological and safety profiles, low toxicity, and some cross the blood-brain barrier efficiently when a suitable route of administration is used. In addition, minocycline and doxycycline have neuroprotective properties in different neurodegenerative diseases [14] due to their anti-inflammatory and anti-apoptotic effects.

Our studies indicated that tetracyclines are good candidate anti-prion drugs. Their ability to bind to fibrillary assemblies, monomeric and oligomeric forms of PrP peptides, homologous to residues 82 to 146 (PrP82-146) and 106 to 126 (PrP106-126) of human PrP, and disrupt peptide aggregates, was previously reported [13]. NMR spectroscopy indicated that tetracyclines achieved through-space interactions with the hydrophobic peptide domains. In cell-free studies the various tetracyclines analogues showed marked differences in facilitating proteinase K (PK) digestion of PrP peptides [15], [7]. In cell culture studies tetracyclines inhibited neuronal death and astroglial proliferation induced *in vitro* by the PrP peptides [13]. Incubation of 263K scrapie-infected brain homogenate with 1 mM tetracycline or doxycycline resulted in more than 90% reduction in the PK-resistant core of PrP^Sc^. It was also reported [16] that these compounds can interact with partially purified PrP^Sc^ from patients with the new variant of Creutzfeldt-Jakob disease, and cattle with bovine spongiform encephalopathy.

*In vivo* there was significant delay in the onset of clinical signs of disease and prolonged survival in hamsters injected with 1 mM tetracycline-pre-treated inocula. When tetracyclines were preincubated with highly diluted scrapie-infected inocula one third of animals did not develop disease [16]. At the time of the onset of symptoms in controls PrP^Sc^ accumulation was less abundant in the brain of tetracycline-treated animals, as was the severity of spongiform changes and astrogliosis in the cerebral cortex and subcortical gray structures [16].

We report the efficacy of tetracycline, doxycycline and minocycline in prolonging the survival of hamsters infected intramuscularly (im), subcutaneously (sc) or intracerebrally (ic) with the 263K scrapie strain and showed that the drugs are also active when administered at the appearance of the first symptoms.

## Results

### Effect of tetracyclines following peripheral scrapie infection

Hamsters were peripherally infected by injecting the 263K scrapie inocula im and sc at 10^−4^ dilution. The im approach was followed to simulate a iatrogenic route of infection and the sc route to slow the spread of scrapie from the periphery to the central nervous system. A high dilution of the 263K scrapie strain was used to ensure a long enough interval to test the effectiveness of the drugs to interfere with scrapie propagation. A high titre of infectivity resulting from an administration route facilitating neuroinvasion would have left little room for any practical *in vivo* screening of anti-prion drugs.

The first experiment was designed to reconfirm our previous observations that tetracycline and doxycycline bind selectively to PrP^Sc^ and sensitize it to protease hydrolysis [16]. Animals were infected im and received a single dose of 10 mg/kg of doxycycline at the same site within 1 hour. Immunohistochemical analysis in preliminary experiments on im infected terminal animals indicated that PrP^Sc^ was evenly distributed in the central nervous and lymphoreticular systems.

**Figure 1:**
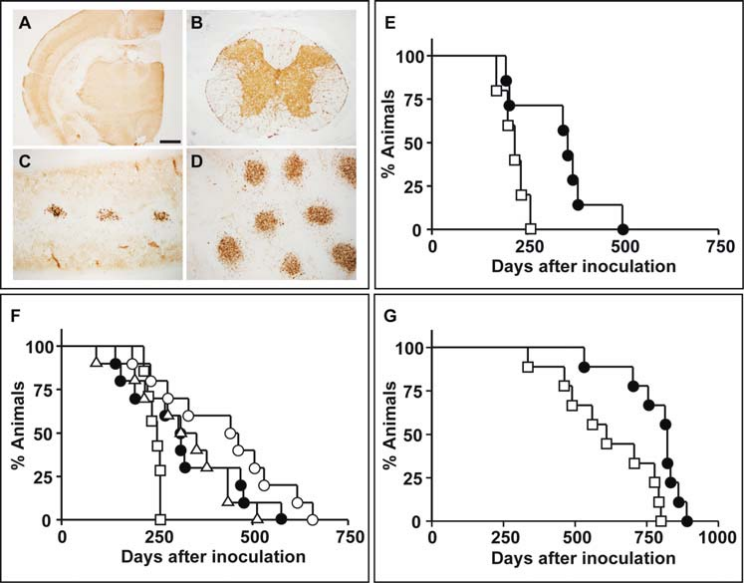
Effect of tetracyclines following peripheral scrapie infection. PrP^Sc^ immunohistochemistry of brain tissue (A), spinal cord (B), spleen (C), and Peyer's patches (D) of hamsters at the terminal stage of disease after intramuscular inoculation of 263K scrapie infected brain homogenate alone at 10^−4^ dilution. All samples were counterstained with hematoxylin. The pictures are representative of the immunohistochemical results for all terminal animals. Magnification scale bar: 1 mm (A), 250 µm (B-C-D). (E) Survival of hamsters injected intramuscularly with a 10^−4^ dilution of 263K scrapie-infected brain homogenate followed one hour later by a single intramuscular dose of doxycycline (10 mg/kg) at the same site. Untreated animals (□), Doxycycline (•). (F) Survival of hamsters injected intramuscularly with a 10^−4^ dilution of 263K scrapie-infected brain homogenate followed one hour later by an intraperitoneal dose of 10 mg/kg of tetracycline, doxycycline or minocycline, then every 2 days up to 40 days post-infection. Untreated animals (□), Tetracycline (▵), Doxycycline, (•), Minocycline (○). (G) Survival of hamsters injected subcutaneously with a 10^−4^ dilution of 263K scrapie-infected brain homogenate followed four days later by an intraperitoneal dose of 10 mg/kg of doxycycline, then every 2 days up to 44 days post-infection. Untreated animals (□), Doxycycline, (•).

As reported in Figure 1 PrP^Sc^ accumulated in all brain areas examined, except for the hippocampus (panel A), in spinal cord (panel B), in the white pulp of spleen (panel C) and in the Peyer's patches (panel D). Figure 1E and Table 1 show that a single dose of doxycycline significantly (p = 0.031) increased median survival by 64%, from 217 days for controls to 355 days for the treated group. This protective effect was paralleled by the delayed onset of clinical signs of disease in all treated animals (data not shown).

**Table 1:** Tetracycline treatment schedules and survival of hamsters infected with 263K scrapie strain.

Infection site	Drugs	Treatment site	Schedule	Median survival (days)	Increase in median survival (days-%)	Hazard ratio**	p value
Intramuscular				217			
Intramuscular	Doxycycline	Intramuscular	10 mg/kg, 1 hour after infection	355	138-64	2.95 (1.17–28.48)	0.031
Intramuscular				250			
Intramuscular	Doxycycline	Intraperitoneal	10 mg/kg, 1 hour after infection and every 2 days for 40 days	312	62-25	2.43 (1.13–15.70)	0.032
Intramuscular	Tetracycline	Intraperitoneal	10 mg/kg, 1 hour after infection and every 2 days for 40 days	330	80-32	2.49 (1.17–16.29)	0.028
Intramuscular	Minocycline	Intraperitoneal	10 mg/kg, 1 hour after infection and every 2 days for 40 days	453	203-81	3.37 (1.98–34.10)	0.004
Subcutaneous				611			
Subcutaneous	Doxycycline	Intraperitoneal	10 mg/kg, 4 days after infection and every 2 days for 44 days	823	212-35	3.14 (1.73–19.10)	0.004
Intracerebral				122			
Intracerebral	LipoDoxycycline	Intracerebroventricular	25 µg/20 µl 30 days after infection	134	12–9.8	4.624 (1.231–17.37)	0.023
Intracerebral				130			
Intracerebral	LipoDoxycycline	Intracerebroventricular	25 µg/20 µl 60 days after infection*	140.5	10–8.1	2.34 (1.08–13.26)	0.037
Intracerebral	LipoMinocycline	Intracerebroventricular	25 µg/20 µl 60 days after infection*	144	14-10	2.10 (0.94–12.31)	0.063

Liposomes containing doxycycline or minocycline (LipoDoxycycline and LipoMinocycline) were prepared as described in Methods.

* At this time the clinical symptoms of disease appeared in 50% of animals.

** In brackets the 95% confidence interval of the hazard ratio.

After this first experiment we designed studies to investigate the effectiveness of intraperitoneal (ip) tetracyclines in animals infected im. Tetracycline, doxycycline and minocycline were administered to hamsters one hour after infection then every two days for 40 days. Figure 1F and Table 1 show that both tetracycline and doxycycline significantly prolonged the median survival of hamsters, by 32% and 25 %, respectively; infected hamsters had a median survival of 250 days while tetracycline and doxycycline increased median survival to 330 (p = 0.028) and 312 days (p = 0.032) respectively. A remarkable result was obtained with minocycline that increased median survival by 81%, from 250 to 453 days (p = 0.004).

A protective effect was also observed in hamsters inoculated sc with 263K scrapie strain homogenate and treated with 10 mg/kg ip doxycycline from day 4 to day 44. In this experiment doxycycline was given four days after the 263K inoculum because of the less efficient spread of infection through subcutaneous mucous membranes. Figure 1G and Table 1 show that doxycycline increased median survival by 35%, with a significant increase of median survival, from 611 to 823 days (p = 0.004).

### Effect of tetracyclines following intracerebral scrapie infection

**Figure 2:**
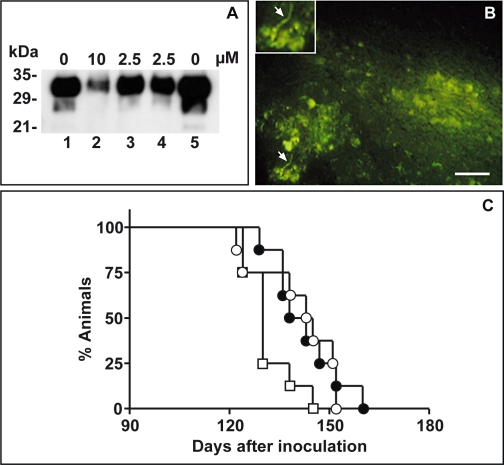
Effect of tetracyclines following intracerebral scrapie infection. (A) Immunoblot analysis of 263K scrapie infected brain homogenate (1%) after incubation in the absence (lane 1), or presence of doxycycline (lanes 2, 3), liposome-containing doxycycline (lane 4) and empty liposomes (lane 5), followed by proteinase K digestion. The blots were probed with the antibody 3F4 (1∶5,000). Molecular mass markers are indicated to the left. (B) Cerebral cortex of hamster four days after an intracerebroventricular infusion of 25 µg/20 µl liposome-containing doxycycline. Doxycycline-related fluorescence appears to be partly diffused in the neuropil and partly associated with nerve cell bodies and processes (arrows) and glial cells. Magnification scale bar: 50 µm. (C) Survival of hamsters injected intracerebrally with a 10^−4^ dilution of 263K scrapie-infected brain homogenate followed 60 days later by a single intracerebroventricular infusion of 25 µg/20 µl doxycycline or minocycline-containing liposomes. By this time 50% of infected animals showed initial clinical symptoms of disease, i.e. hyperreactivity to tactile and acoustic stimulations. Untreated animals (□), LipoDoxycycline, (•), LipoMinocycline (○).

In the light of the results after peripheral infection we set up an experiment to verify whether tetracyclines prolonged the lifespan of animals at the onset of clinical symptoms. We tested a novel approach for tetracycline administration which permitted a more aggressive treatment schedule in a model of overt prion disease, i.e. ic inoculation of the 263K scrapie strain followed by intracereboventricular (icv) infusion of liposome-entrapped drugs. To overcome the risk of toxicity caused by icv administration of tetracycline solutions we entrapped the drugs in liposomes. In preliminary studies liposome-entrapped tetracyclines and empty liposomes showed no toxicity, indicating that icv infusion could be used to deliver high local drug concentrations (data not shown). We also verified the effectiveness of doxycycline-entrapped liposomes (LipoDoxycycline) to reverse PrP^Sc^ resistance to PK action. Figure 2A shows that incubation of the 263K scrapie strain homogenate with LipoDoxycycline for 24 hours increased the sensitization to PK hydrolysis to the same extent as an identical concentration of the free molecule. As expected, empty liposomes had no such effect.

We also verified that tetracyclines diffuse into the parenchyma after liposome infusion into the lateral ventricle. To this end, 20 µl of liposomes containing 25 µg doxycycline were infused icv and the brain distribution of the drug was determined four days later by fruorescence microscopy of frozen sections. Doxycycline-related fluorescence was intense in correspondence with the ependymal cell layer and subependymal space of the ventricular system, the leptomeninges and subpial space, and in some meningeal and parenchymal vessel walls and perivascular space. Noteworthy, doxycycline fluorescence was also present in brain parenchyma, partly diffused in the neuropil and partly associated with nerve cell bodies and processes and glial cells (Figure 2B). No significant fluorescence signals were detected in the brain injected with empty liposomes.

To explore whether the time of intervention is important in delaying the disease, in a first experiment LipoDoxycycline (25 µg/20 µl, icv) was given 30 days after prion inoculation. This treatment schedule caused a significant (p = 0.023) increase of the median survival of 9.8% (from 122 to 134 days), indicating that the therapeutic intervention could in fact be postponed (Table 1). To investigate whether tetracyclines were effective at the onset of the clinical symptoms associated with prion infection LipoDoxycycline or LipoMinocycline (25 µg/20 µl) were infused icv 60 days after ic infection with the 263K scrapie strain when 50% of the animals showed initial symptoms of disease (hyperreactivity to tactile and acoustic stimulation). Figure 2E and Table 1 show that both preparation increased the median survival respectively by 8.1% (from 130 to 140.5 days, p = 0.037) and 10% (from 130 to 144 days, p = 0.063).

## Discussion

We have previously shown that the mechanism of action of tetracyclines as anti-prion drugs depends on their effectiveness in sensitizing PrP^Sc^ to proteolytic degradation, after binding to lypophilic domains of the protein, reducing or abolishing prion infectivity [13], [21]. Tetracyclines bind not only to PrP aggregates but also to neurotoxic peptides, antagonizing their toxic effect on nerve and glial cells, and inhibiting astroglial proliferation [13]. In these last years tetracyclines have been found to inhibit *in vitro* aggregation and disrupt amyloid fibrils of a variety of other fibrillogenic proteins, suggesting they may be effective in other disorders related to misfolded proteins [7], [22]–[27]. They exert beneficial effects *in vivo* in models of Parkinson's, Huntington's and Alzheimer's diseases, by inhibiting caspase-1, caspase-3, inducible nitric oxide synthase expression and nitric oxide-mediated toxicity, although a role of their anti-fibrillogenic efficacy cannot be ruled out [28]–[30]. In these models minocycline was particularly effective, crossing the BBB more than the other tetracyclines. However, in a model of familial amyloidotic polyneuropathy, doxycycline disaggregated the deposits of extracellular fibrils of transthyretin in mice [31].

This study comprised peripheral (intramuscular and subcutaneous) and intracerebral administration of the 263K scrapie strain to mimic respectively iatrogenic and sporadic forms of the disease. The intramuscular route of infection was very efficient in spreading the disease in the animals, confirming that skeletal muscle has a preferential network in the propagation of the infection. For instance, in our laboratory the median survival of hamsters treated with the same homogenate at the dilution of 10^−4^ of 263 K scrapie strain was respectively 259, 234, 122 days after ip, im and ic injection. Subcutaneous inoculation of the 263 K scrapie strain was used as a model of penetration of PrP^Sc^ through the skin mucous membranes. Subcutaneous transmission of PrP^Sc^ efficiently spreads the disease in immunocompetent animals through follicular dendritic cells in the epidermis [34]. In our experimental model the latency of the disease was much longer (median survival 611 days in control animals) compared to the other routes of administration. For long-term ip treatments we have used a dose of 10 mg/kg/day which is well tolerated by hamsters [32], [33]. Higher dose levels of 20 or 25 mg/kg/day could not be used due to manifestation of overt toxicity and serious inflammatory effects at the site of injection.

The effectiveness of tetracyclines after im and sc infection in prolonging the life-span of animals suggests they may be useful candidates for prevention strategies of acquired forms of the disease.

Intracerebroventricular administration of quinacrine, amphotericin B, pentosan polysulfate and quinoline derivatives in transgenic mice inoculated ic with 263K, RML and Fukuoka 1 strains was carried out by Doh-ura et al. [35] and Murakami-Kubo et al. [36] in an experimental model of prolonged infusion lasting four weeks. The treatment with amphotericin B or pentosan polysulfate was effective when started in early stages of the disease and quinine had efficacy when given in a relatively late stage of the disease.

We investigated the effectiveness of doxycycline and minocycline at an advanced stage of infection, i.e. 60 days after inoculation of 263 K scrapie strain homogenate, when 50% of animals showed the first neurological symptoms. A single infusion of 25 µg/20 µl icv of tetracyclines entrapped in liposomes was used since infusion of tetracycline solutions can induce overt acute toxicity. This enabled us to reach doxycycline and minocycline levels of 50 µg/g in brain, well above the Cmax that can be measured in rats after 25 mg/kg of minocycline iv [19]. The concentration of tetracycline in liposomes infused icv was the highest possible based upon the entrapment recovery of the drugs. A single icv infusion of LipoDoxycycline or LipoMinocycline at the onset of the first neurological symptoms increased survival, reaching statistical significance for doxycycline and close to significance for minocycline. In our opinion this difference, however, reflects the small number of animals in each experimental group, which reduced the statistical power of the analysis, and the lower effectiveness of minocycline in sensitizing PrP peptides to protease hydrolysis, as shown in cell-free tests [7]. Moreover, the comparable efficacy of icv LipoDoxycycline or LipoMinocycline indicates that the BBB is crucial in the survival of animals treated ip. Minocycline, which is known to cross the BBB to a greater extent than tetracycline and doxycycline, was the most effective molecule.

Our data confirm that liposomes containing tetracyclines can be safely administered icv and suggest that continuous infusions could improve their therapeutic index. The development of pharmaceutical formulations employing liposomes or nanoparticles to facilitate the BBB passage of these drugs might offer alternative ways of administration such as continuous iv or ip infusions to attain high steady-state drugs levels in the brain.

The broad efficacy of tetracyclines has to be confirmed in different animal models and with different prion strains. In fact, the onset and progression of the disease is variable and highly dependent on the prion strain and host animal species considered. The host/prion strain interaction is regulated by multiple factors, such as accumulation and distribution of PrPSc, incubation time and length of the disease as well as protein sensitivity to proteinase K degradation [37], [38]. The last factor is likely to be crucial in dictating sensitivity to tetracyclines, induction of proteinase K-dependent degradation of PrP^Sc^ is purported to be the major mechanism underlying the anti-prion activity of these drugs. In this context, it is worth mentioning that the antibiotic Amphotericin B, which is known to decrease resistance to proteinase K digestion, prolongs the lifespan of hamsters infected with the 263K strain of PrP^Sc^ [39], [40]. However, its therapeutic activity is limited to this prion strain and is not observed after infection of the animals with other strains [41]. Ongoing studies are aimed at verifying the effectiveness of tetracycline treatment in hamsters and mice using PrP^Sc^ strains with different propagation properties and sensitivity to proteinase K action.

A further point worth exploring is the dissociation between the anti-microbial and anti-fibrillogenic activity of tetracyclines. Formally we have not excluded the possibility that the beneficial effect of tetracyclines may results from other as yet unresolved mechanisms. Though unlikely, it is possible that the increased survival of the animals is related to antibiotic activity of tetracyclines. As the chemical functionalities responsible of the antibiotic activity of these drugs are known [42] we are in the process of testing the anti-prion properties of tetracyclines devoid of anti-microbial activity.

In conclusion this study found that tetracyclines can counteract the onset and progression of prion disease and might therefore be potentially useful for human therapy. In the last five years, a small group of CJD patients has received compassionate treatment with daily doses of 100 mg/kg doxycycline, and retrospective analysis showed significantly longer survival than untreated patients (Tagliavini F, et al., manuscript in preparation). Once again whether this effect is related to anti-prion activity or to protection of patients from bacterial infection is yet to be established. A major limitation of this study is that the results are not the outcome of a formal clinical trial but are based on open observations. The data reported in this paper provided the experimental basis for an ongoing Italian phase II, multicenter, randomized, double-blind, placebo-controlled efficacy study of doxycycline in CJD patients funded by the Italian Drug Agency.

## Materials and Methods

### Prion transmission

Male Golden Syrian hamsters (Charles River, Calco, Lecco, Italy), 6–8 weeks old, were im (in the posteriour leg) or sc (under the skin of the back) infected with 100 µl of 263K scrapie strain at a 10^−4^ dilution from scrapie-infected hamster brains at the terminal stage of disease. Other hamsters were infected ic with 20 µl of the same scrapie strain. Each experimental group consisted of 10–12 animals.

Tetracycline hydrochloride (Fluka, Switzerland), doxycycline hyclate and minocycline hydrochloride (Sigma Aldrich, Switzerland) were freshly dissolved in saline before ip administration. After prion infection, ten animals per group were treated according to the schedules summarized in Table 1. Doxycycline was injected im, 10 mg/kg, 1 hour after im prion infection. Doxycycline, minocycline or tetracycline were injected ip, 10 mg/kg, 1 hour after im prion infection then every two days for 40 days, or from 4 to 44 days after sc prion infection. Ic infected hamsters were anesthetized with chloral hydrate (Merck, Germany) and a polyethylene cannula was permanently implanted into the lateral ventricle opposite to the hemisphere injected with 263K brain homogenate. Then a suspension 20 µl of liposomes containing 25 µg of doxycycline or minocycline were infused icv when the first symptoms of infection appeared (60 days) in 50% of hamsters (hyperreactivity to tactile and acoustic stimulation). Non-infected control animals were treated with liposomes containing tetracyclines.

Animals were housed in groups of 3–4 in a temperature-controlled (20±2°C) quarantined room maintained on a 12-hour light-dark cycle. They were allowed free access to food and water and were observed once a week until terminal stage of the disease to record the onset and progression of clinical signs. Behavioural analysis included evaluation of their reactivity to tactile and acoustic stimulation, posture, balance and coordination, and the onset of tremors [17].

Procedures involving animals and their care were conducted in conformity with national and international laws and policies (EEC Council Directive 86609, OJ L358, 1, 12 December 1987; Italian Legislative Decree 116/92, Gazzetta Ufficiale della Repubblica Italiana n.10, 18 February 1992; Guide for the Care and Use of Laboratory Animals, US National Research Council, 1996).

### Preparation of liposome-containing tetracyclines

Doxycycline and minocycline were entrapped in neutral multilamellar liposomes (LipoDoxycycline and LipoMinocycline) made up of phosphatidylcholine and cholesterol (7∶1, molar ratio) [18]. Empty liposomes were made of phosphatidylcholine and cholesterol alone. The concentration of drug entrapped in liposomes was determined by HPLC [19] and the entrapment efficiency was 5% for both tetracyclines (mean of five preparations). Liposomes were stable for at least two weeks at 4°C in the dark.

### Brain distribution of liposome-containing doxycycline

To determine the brain distribution of LipoDoxycycline and empty liposomes, hamsters were icv treated as described above and killed four days later. Brains were dissected and 20 µm cryostat sections were prepared. No significant fluorescence signals were detected in the brain injected with empty liposomes. Drug distribution was evaluated by determining doxycycline fluorescence in coronal sections with a microscope (Olympus BX51) at 400–440 nm excitation and 475 nm emission.

### Reversal of PrP^Sc^ protease-resistance by liposome-containing doxycycline

To investigate whether LipoDoxycycline was as effective as free doxycycline during the PrP^Sc^ folding, 1% 263K brain homogenate (10^−2^ dilution) was incubated for 24 hours at 37°C in the presence of doxycycline (25–100 µg), LipoDoxycycline (25 µg) or the same volume of empty liposomes. Samples were then treated for 1 hour at 37°C with 10 µg/ml of PK (Roche, Switzerland). The amount of PrP^Sc^ remaining after proteolysis was assessed by Western blot analysis as described by Tagliavini et al. [13].

### Immunohistochemical studies

At autopsy, the left lateral two-third of each hamster brain, one half of the spleen, a fraction of the small intestine at the level of Peyer's patches and the spinal cord were dissected and immediately fixed for 24 hours in Carnoy solution at 4°C [20]. The fixed brains were then dissected at four standard coronal levels. All fixed tissues were dehydrated, embedded in Paraplast and 5-µm thick serial sections were stained with hematoxylin-eosin. PrP^Sc^ immunohistochemistry was carried out using monoclonal antibody 3F4 (1∶1000 dilution, Dako, Denmark) which recognized an epitope including residues 109 to 112 of hamster PrP. Before immunostaining, sections were treated with 10 µg/ml of PK for 5 min then with 3M guanidine thiocyanate for 30 min [20]. Immunoreactions were revealed with the monoclonal EnVision system (Dako, Denmark) using 3-3′-diamonobenzidine as chromogen.

### Statistical analysis

Survival times were analyzed by Kaplan-Meier Survival Analysis using the log-rank test to compare the curves. Statistical analysis was done using Prism version 4.0 for Windows (GraphPad Software, San Diego, CA).

## References

[1] Prusiner SB (2001). Shattuck lecture–neurodegenerative diseases and prions.. N Engl J Med.

[2] Prusiner SB (1998). Prions.. Proc Natl Acad Sci U S A.

[3] Riesner D (2003). Biochemistry and structure of PrP(C) and PrP(Sc).. Br Med Bull.

[4] Korth C, Peters PJ (2006). Emerging pharmacotherapies for Creutzfeldt-Jakob disease.. Arch Neurol.

[5] Caramelli M, Ru G, Acutis P, Forloni G (2006). Prion diseases: current understanding of epidemiology and pathogenesis, and therapeutic advances.. CNS Drugs.

[6] Weissmann C, Aguzzi A (2005). Approaches to therapy of prion diseases.. Annu Rev Med.

[7] Forloni G, Varì MR, Colombo L, Bugiani O, Tagliavini F (2003). Prion disease: time for a therapy ?. Curr Med Chem-Immun, Endoc & Metab Agents.

[8] Todd NV, Morrow J, Doh-ura K, Dealler S, O'Hare S (2005). Cerebroventricular infusion of pentosan polysulphate in human variant Creutzfeldt-Jakob disease.. J Infect.

[9] Rainov NG, Tsuboi Y, Krolak-Salmon P, Vighetto A, Doh-ura K (2007). Experimental treatments for human transmissible spongiform encephalopathies: is there a role for pentosan polysulfate?. Expert Opin Biol Ther.

[10] Nakajima M, Yamada T, Kusuhara T, Furukawa H, Takahashi M (2004). Results of quinacrine administration to patients with Creutzfeldt-Jakob disease.. Dement Geriatr Cogn Disord.

[11] Benito-Leon J (2004). Combined quinacrine and chlorpromazine therapy in fatal familial insomnia.. Clin Neuropharmacol.

[12] Otto M, Cepek L, Ratzka P, Doehlinger S, Boekhoff I (2004). Efficacy of flupirtine on cognitive function in patients with CJD: A double-blind study.. Neurology.

[13] Tagliavini F, Forloni G, Colombo L, Rossi G, Girola L (2000). Tetracycline affects abnormal properties of synthetic PrP peptides and PrP (Sc) in vitro.. J Mol Biol.

[14] Domercq M, Matute C (2004). Neuroprotection by tetracyclines.. Trends Pharmacol Sci.

[15] Cosentino U, Varì MR, Saracino AA, Pitea D, Moro G (2005). Tetracycline and its analogues as inhibitors of amyloid fibrils: searching for a geometrical pharmacophore by theoretical investigation of their conformational behavior in aqueous solution.. J Mol Model.

[16] Forloni G, Iussich S, Awan T, Colombo L, Angeretti N (2002). Tetracyclines affect prion infectivity.. Proc Natl Acad Sci U S A.

[17] Tagliavini F, McArthur RA, Canciani B, Giaccone G, Porro M (1997). Effectiveness of anthracycline against experimental prion disease in Syrian hamsters.. Science.

[18] Konings AWT (1984). Lipid peroxidation in liposome. In: Gregoriadis G, ed. Liposome technology..

[19] Colovic M, Caccia S (2003). Liquid chromatographic determination of minocycline in brain-to-plasma distribution studies in the rat.. J Chromatogr B Analyt Technol Biomed Life Sci.

[20] Giaccone G, Canciani B, Puoti G, Rossi G, Goffredo D (2000). Creutzfeldt-Jakob disease: Carnoy's fixative improves the immunohistochemistry of the proteinase K-resistant prion protein.. Brain Pathol.

[21] Ragg E, Tagliavini F, Malesani P, Monticelli L, Bugiani O (1999). Determination of solution conformations of PrP106-126, a neurotoxic fragment of prion protein, by 1H NMR and restrained molecular dynamics.. Eur J Biochem.

[22] Malmo C, Vilasi S, Iannuzzi C, Tacchi S, Cametti C (2006). Tetracycline inhibits W7FW14F apomyoglobin fibril extension and keeps the amyloid protein in a pre-fibrillar, highly cytotoxic state.. FASEB J.

[23] Ono K, Yamada M (2006). Antioxidant compounds have potent anti-fibrillogenic and fibril- destabilizing effects for alpha-synuclein fibrils in vitro.. J Neurochem.

[24] Aitken JF, Loomes KM, Konarkowska B, Cooper GJ (2003). Suppression by polycyclic compounds of the conversion of human amylin into insoluble amyloid.. Biochem J.

[25] Cardoso I, Merlini G, Saraiva MJ (2003). 4′-iodo-4′-deoxydoxorubicin and tetracyclines disrupt transthyretin amyloid fibrils in vitro producing noncytotoxic species: screening for TTR fibril disrupters.. FASEB J.

[26] Smith DL, Woodman B, Mahal A, Sathasivam K, Ghazi-Noori S (2003). Minocycline and doxycycline are not beneficial in a model of Huntington's disease.. Ann Neurol.

[27] Forloni G, Colombo L, Girola L, Tagliavini F, Salmona M (2001). Anti-amyloidogenic activity of tetracyclines: studies in vitro.. FEBS Lett.

[28] Du Y, Ma Z, Lin S, Dodel RC, Gao F (2001). Minocycline prevents nigrostriatal dopaminergic neurodegeneration in the MPTP model of Parkinson's disease.. Proc Natl Acad Sci U S A.

[29] Hersch S, Fink K, Vonsattel JP, Friedlander RM (2003). Minocycline is protective in a mouse model of Huntington's disease.. Ann Neurol.

[30] Seabrook TJ, Jiang L, Maier M, Lemere CA (2006). Minocycline affects microglia activation, Abeta deposition, and behavior in APP -tg mice.. Glia.

[31] Cardoso I, Saraiva MJ (2006). Doxycycline disrupts transthyretin amyloid: evidence from studies in a FAP transgenic mice model.. FASEB J.

[32] Moon JE, Ellis MW, Griffith ME, Hawley JS, Rivard RG (2006). Efficacy of macrolides and telithromycin against leptospirosis in a hamster model.. Antimicrob Agents Chemother.

[33] Truccolo J, Charavay F, Merien F, Perolat P (2002). Quantitative PCR assay to evaluate ampicillin, ofloxacin, and doxycycline for treatment of experimental leptospirosis.. Antimicrob Agents Chemother.

[34] Mohan J, Bruce ME, Mabbott NA (2005). Follicular dendritic cell dedifferentiation reduces scrapie susceptibility following inoculation via the skin.. Immunology.

[35] Doh-ura K, Ishikawa K, Murakami-Kubo I, Sasaki K, Mohri S (2004). Treatment of transmissible spongiform encephalopathy by intraventricular drug infusion in animal models.. J Virol.

[36] Murakami-Kubo I, Doh-Ura K, Ishikawa K, Kawatake S, Sasaki K (2004). Quinoline derivatives are therapeutic candidates for transmissible spongiform encephalopathies.. J Virol.

[37] Clarke AR, Jackson GS, Collinge J (2001). The molecular biology of prion propagation.. Philos Trans R Soc Lond B Biol Sci..

[38] Bruce ME (2003). TSE strain variation.. Br Med Bull.

[39] Demaimay R, Adjou K, Lasmézas C, Lazarini F, Cherifi K, Seman M, Deslys JP, Dormont D (1994). Pharmacological studies of a new derivative of amphotericin B, MS-8209, in mouse and hamster scrapie.. J Gen Virol.

[40] Adjou KT, Privat N, Demart S, Deslys JP, Seman M, Hauw JJ, Dormont D (2000). MS-8209, an amphotericin B analogue, delays the appearance of spongiosis, astrogliosis and PrPres accumulation in the brain of scrapie-infected hamsters.. J Comp Pathol.

[41] Demaimay R, Race R, Chesebro B (1999). Effectiveness of polyene antibiotics in treatment of transmissible spongiform encephalopathy in transgenic mice expressing Syrian hamster PrP only in neurons.. J Virol.

[42] Chopra I, Roberts M (2001). Tetracycline antibiotics: mode of action, applications, molecular biology, and epidemiology of bacterial resistance.. Microbiol Mol Biol Rev.

